# Body Mass Index and Risk of Parkinson’s Disease: A Dose-Response Meta-Analysis of Prospective Studies

**DOI:** 10.1371/journal.pone.0131778

**Published:** 2015-06-29

**Authors:** Yun-Liang Wang, Yu-Tong Wang, Jin-Feng Li, Yu-Zheng Zhang, Hong-Lei Yin, Bing Han

**Affiliations:** 1 Department of Neurology, the 148 Central Hospital of PLA, Zibo, 255000, China; 2 School of Medicine, Henan University, Kaifeng, 475000, China; Weill Cornell Medical College in Qatar, QATAR

## Abstract

**Background:**

A number of epidemiologic studies examining the relationship between body mass index (BMI) and the future occurrence of Parkinson’s disease (PD) reported largely inconsistent findings. We conducted a dose-response meta-analysis of prospective studies to clarify this association.

**Methods:**

Eligible prospective studies were identified by a search of PubMed and by checking the references of related publications. The generalized least squares trend estimation was employed to compute study-specific relative risks (RR) and 95% confidence intervals (CI) for an increase in BMI of 5 kg/m^2^, and the random-effects model was used to compute summary RR and 95% CI.

**Results:**

A total of 10 prospective studies were included in the final analysis. An increase in BMI of 5 kg/m^2^ was not associated with PD risk, with a summary RR of 1.00 (95% CI = 0.89-1.12). Results of subgroup analysis found similar results except for a week positive association in studies that adjusted for alcohol consumption (RR = 1.13, 95% CI = 0.99-1.29), and a week inverse association in studies that did not (RR = 0.90, 95% CI = 0.78-1.04). In a separate meta-analysis, no significant association between overweight (25 kg/m^2^ ≤ BMI ≤29.9 kg/m^2^), obesity (BMI≥30 kg/m^2^) or excess weight (BMI≥25 kg/m^2^) and PD risk was observed.

**Conclusion:**

This meta-analysis does not support the notion that higher BMI materially increases PD risk. However, a week positive BMI-PD association that may be masked by confounders still cannot be excluded, and future prospective studies with a good control for potential confounding factors are needed.

## Introduction

Obesity is a well-established risk factor for several metabolic and vascular disorders such as type 2 diabetes, coronary heart disease and stroke, some of which may be a contributor to Parkinson’s disease (PD)[1, 2]. However, the relationship between obesity and the occurrence of PD is unknown. A recent meta-analysis[3] of 12 case-control studies showed that PD patients had a significant lower BMI than controls. Nonetheless, patients with PD may have started to lose weight prior to the clinical diagnosis [4, 5], and a lower BMI in PD patients does not represent an inverse BMI-PD relationship. A very recent meta-analysis[6] including 3 case-control studies and 4 prospective studies reported that overweight (defined as 25 kg/m^2^≤BMI<30 kg/m^2^), but not obesity (BMI≥30 kg/m^2^) was associated with increased risk of PD. However, only crude risk estimates (without adjustment for potential confounders) were pooled, and the reported results may not reflect the true magnitude of the association. Taking potential confounders, such as smoking, alcohol and coffee consumption, and physical activity is of particular importance because they may be related to both BMI and PD[7–12]. In addition, there are several other prospective studies[5, 7, 10, 13, 14] that have not been included in the meta-analysis. To update the epidemiological evidence and clarify the relationship between BMI, or obesity status measured by BMI levels, and future risk of PD, we performed this dose-response meta-analysis of prospective studies.

## Materials and Methods

### Selection criteria

We searched for potentially relevant publications on PubMed database from 1966 through December, 2014 using the following search strategy: (overweight OR access weight OR obesity OR obese OR adiposity OR body mass index) and (Parkinson disease OR Parkinson’s disease). In order to identify any further studies, the references cited by retrieved full texts, related reviews and previous meta-analyses were also carefully checked. Eligible studies were prospective studies that reported relative risks (RR) and 95% confidence intervals (CI) (or data to estimate them) for BMI (in kg/m^2^), or obesity status measured by BMI, in relation to incidence of PD.

### Data extraction

For each eligible study included, the following characteristics were collected: the first author’s last name, year of publication, country, study name, years of follow-up, sex and age of participants, number of cases and participants, detailed levels of BMI, RR estimates with corresponding 95% CIs, and potential confounders adjusted for in the multivariable analyses. Literature selection and data extraction were independently performed by two reviewers (YTW and JFL), and any discrepancies were adjudicated by the third one (YZZ).

### Statistical analysis

The RRs that reflect the greatest degree of control for confounders (if available) were used in the meta-analysis. Due to the distinct cut-off points for categories across studies, we estimated a RR with 95% CI for a 5 kg/m^2^ increase in BMI for each study, if the study only reported results by categories of BMI. The method of generalized least squares trend estimation proposed by Greenland and Longnecker [15] and Orsini *et al*.[16] was employed to compute study-specific slopes and 95% CIs from the correlated logs of the RRs and CIs across BMI categories. Accordingly, the levels of BMI, distributions of cases and person-years, and RRs with 95% CIs were extracted from each study. The median or mean levels of BMI in each category were used as the average levels. If the median or mean value per category was not provided, the midpoint of the upper and lower boundaries was considered average levels. If the highest or lowest category was open-ended, we assumed the width of the interval to be the same as in the closest category. Then, we combined study-specific risk estimates with the random-effects model that takes into account both within- and between-study variation [17].

Subgroup analyses were performed according to geographic region, sex, years of follow-up, baseline age (mean or median) of participants, number of cases, and adjustment for potential confounders. A sensitivity analysis in which one study was removed and the rest were analyzed was conducted to assess whether the results were influenced by any single studies. We further conducted a separate meta-analysis by obesity status by creating 3 BMI categories, namely overweight (25 kg/m^2^ ≤ BMI ≤29.9 kg/m^2^), obesity (BMI≥30 kg/m^2^) and excess weight (BMI≥25 kg/m^2^)[18]. For studies that reported RRs for more than one category of BMI that fell into same category, we pooled these RRs with the fixed-effect model and used the pooled estimates in this analysis.

Heterogeneity among studies was assessed with the Q and *I* ^2^ statistics [19]. For the Q statistic, a *P*-value of less than .1 was considered statistically significant heterogeneity. Potential publication bias was evaluated with both Begg rank correlation test and Egger linear regression test [20, 21]. All analyses were performed using STATA12.0 (StataCorp, College Station, TX, USA).

## Results

### Study characteristics

**Fig 1:**
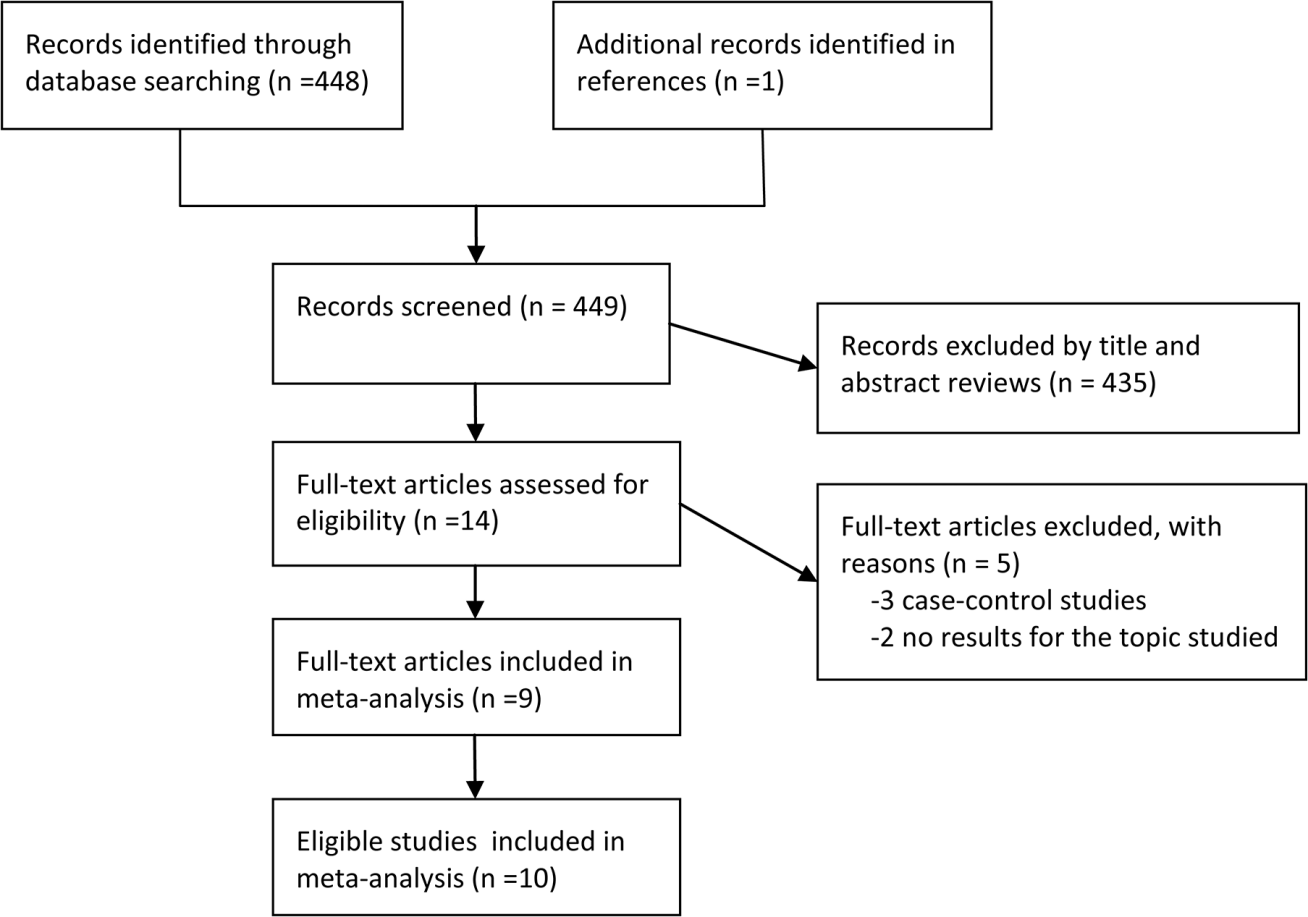
Flow diagram of literature search for the meta-analysis.

A total of 448 records were yielded in the primary search and 1 additional citation was found in reference lists. Fourteen full articles remained after the title and abstract reviewing, of which 5 were excluded for the reasons reported in Fig 1 and S1 Table. Finally, 9 publications [4, 5, 7, 10, 13, 14, 22–24] including 10 independent prospective studies that evaluated the association between BMI and risk of Parkinson’s disease were included in this meta-analysis, with 2,706 PD cases and 430,854 participants involved. These studies were published between 2002 and 2014. Six studies were conducted in the United States, 2 studies were carried out in Finland, 1 study was performed in Greece and 1 was from China. The number of cases ranged from 85 to 556, and the number of participants ranged from 85 to 147,096. Four studies included men only, 1 study recruited women only, and 5 studies involved both men and women. All but 1 study had a length of follow-up of at least 10 years. Two studies did not report risk estimates for the association of BMI with PD, and we calculated crude RR with corresponding 95% CI according to the raw data reported. The levels of BMI, the reported RR with corresponding 95% CI, as well as the variables accounted for in the multivariable analyses varied substantially across individual studies. The characteristics of included studies are summarized in Table 1.

**Table 1:** Characteristics of included prospective studies on the association between body mass index and risk of Parkinson’s disease.

Study	Country	Study name	Years of	Sex	Age at	No.of cases/	BMI levels,	RR (95% CI)	Adjustment
			follow-up		baseline	participants	kg/m^2^		
Abbott, 2002	USA	Honolulu Heart Program	30	M	54	137/7990	14.3–21.7	Ref.	None.
							21.8–23.8	2.06 (1.21–3.50) ^a^	
							23.9–25.8	2.23 (1.32–3.77) ^a^	
							25.9–39.9	1.55 (0.89–2.72) ^a^	
Chen, 2004	USA	Health Professionals	14	M	40–75	249/47700	<23	Ref.	Age, smoking, caffeine intake,
		follow-up study					23–24.9	1.1 (0.8–1.7)	and alcohol consumption.
							25–26.9	1.0 (0.7–1.5)	
							27–29.9	1.1 (0.8–1.7)	
							≥30	1.0 (0.6–1.8)	
Chen, 2004	USA	Nurses’ Health Study	22	F	30–55	202/117062	<23	Ref.	Age and smoking.
							23–24.9	1.0 (0.7–1.5)	
							25–26.9	1.0 (0.7–1.6)	
							27–29.9	0.7 (0.4–1.2)	
							≥30	0.7 (0.4–1.2)	
Hu, 2006	Finland	Finnish cohort	18.8	M/F	25–59	526/45806	<23	Ref.	Age, smoking, physical activity, study
							23–24.9	1.70 (1.23–2.37)	year, systolic BP, cholesterol, eduction,
							25–26.9	1.70 (1.23–2.37)	and alcohol, coffee, and tea consumption
							27–29.9	2.02 (1.46–2.79)	
							≥30	2.03 (1.44–2.85)	
Ma, 2006 ^b^	China	Nutritional	13	M/F	68.25	85/425	<20	Ref.	Age and sex.
		Intervention Trial					20–21.5	0.76 (0.40–1.47)	
							21.5–23	0.88 (0.46–1.66)	
							≥23	0.43 (0.20–0.93)	
Logroscino, 2007	USA	Harvard Alumni	10	M	67.7	106/10812	<22.5	1.51 (0.95–2.40)	Age, smoking, physical activity,
		Health Study					22.5–25.0	Ref.	history of CVD or cancer, and tea and
							≥25.0	0.86 (0.53–1.41)	coffee consumption.
Driver, 2008	USA	Physicians’ Health	23	M	40–84	556/21841	<25	Ref.	None.
		Study					25–30	1.02 (0.86–1.21) ^a^	
							≥30	0.85 (0.54–1.35) ^a^	
Palacios, 2011^b^	USA	Cancer Prevention	13	M/F	63.6 (M)	656/147096	18.5–23	Ref.	Age, smoking, physical activity,
		ⅡNutrition Cohort			62.0 (F)		23–24.9	1.00 (0.78–1.26)	education, pesticide exposure, energy
							25–26.9	0.95 (0.60–1.50)	intake, and alcohol, caffeine, and
							27–29.9	1.11 (0.86–1.42)	dairy consumption.
							≥30	1.00 (0.75–1.34)	
Kyrozis, 2013	Greece	EPIC-Greece	8.45	M/F	20–86	88/25407	Each	0.86 (0.53–1.39)	Age, sex, smoking, physical activity,
							10kg/m^2^		marital status, education, farming, energy
							increase		intake, and coffee consumption.
Saaksjarvi, 2014	Finland	Finish Mobile Clinic	22	M/F	50–79	101/6715	<23	Ref.	Age, sex, smoking, education, physical
		Health Examination					23–24.9	1.04 (0.50–2.17)	activity, community density, occupation,
							25–27.4	0.91 (0.45–1.83)	and coffee and alcohol consumption.
							27.5–29.9	1.46 (0.74–2.87)	
							≥30	1.09 (0.54–2.21)	

BMI, body mass index; BP, blood pressure; CI, confidence interval; CVD, cardiovascular disease; F, female; M, male; RR, relative risk;

^a^ relative risk and 95% confidence interval were estimated using reported original data.

^b^ sex-specific results were also reported in the publications.

### BMI and PD

**Fig 2:**
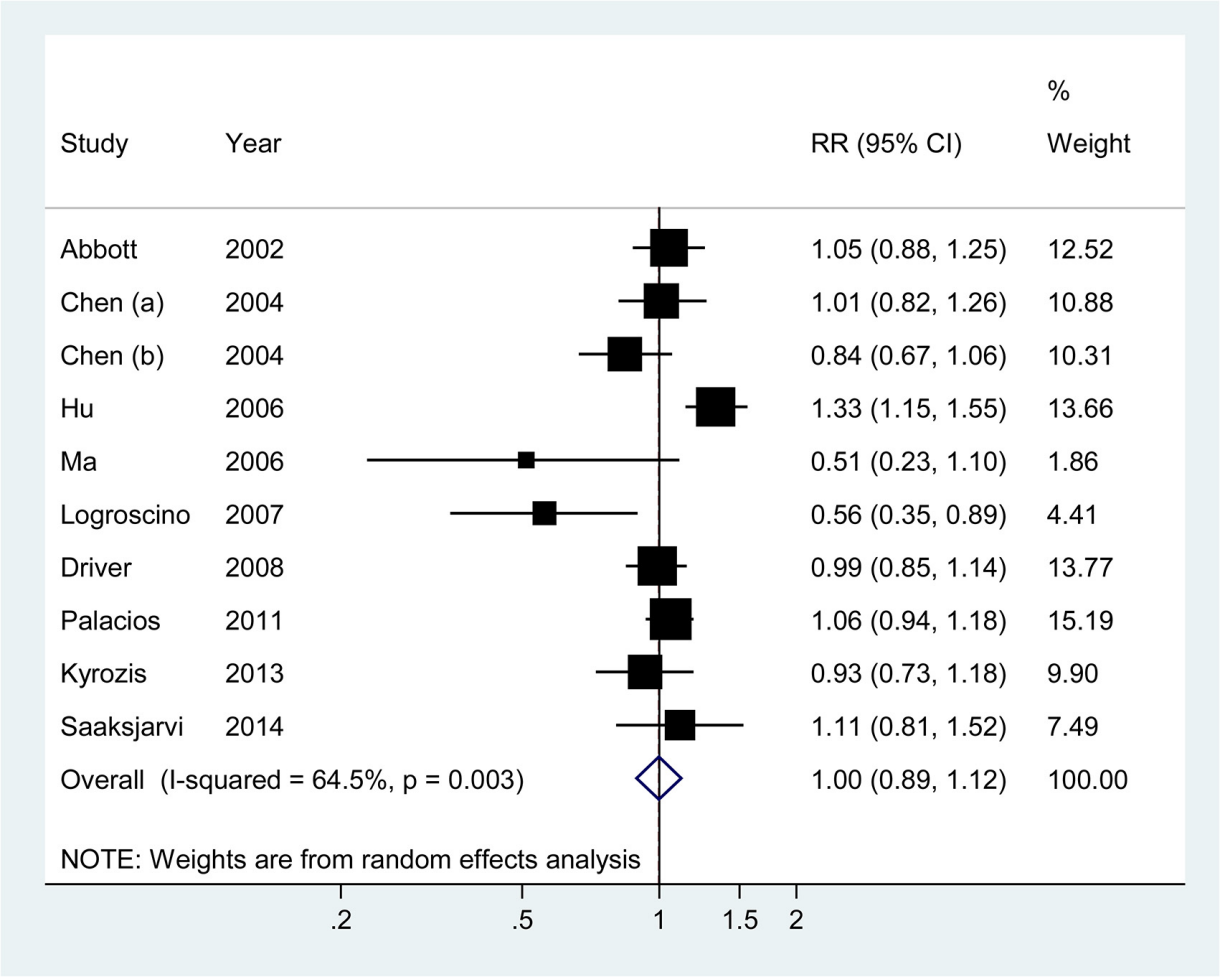
Relative risks and 95% confidence interval of Parkinson’s disease for an increment in body mass index of 5 kg/m^2^ for individual studies and all studies combined.

A meta-analysis of the 10 prospective studies showed a summary RR of 1.00 (95% CI = 0.89–1.12) for an increase in BMI of 5 kg/m^2^ (Fig 2), which suggested that BMI was not associated with risk of PD. There was significant heterogeneity among studies (*P*_heterogeneity_ = 0.003, *I* ^2^ = 64.5%).

### Subgroup analysis and sensitivity analysis

The results of subgroup analysis stratified by geographic region, sex, study duration, age of participants, number of cases, and adjustment for confounders are reported in Table 2. We consistently showed no relationship between BMI and risk of PD in these analyses. There was a suggestion of weekly increased risk of PD in studies that adjusted for alcohol consumption (RR = 1.13, 95% CI = 0.99–1.29), and slightly decreased risk in studies that did not (RR = 0.90, 95% CI = 0.78–1.04), but the difference by meta-regression analysis was statistically non-significant (*P*_differences_ = 0.08). In the sensitivity analyses in which one study was removed and the rest were analyzed, the pooled RR of PD ranged from 0.98 (95% CI = 0.86–1.12) to 1.03 (95% CI = 0.93–1.14).

**Table 2:** Subgroup for the association between body mass index (in increments of 5 kg/m^2^) and risk for Parkinson’s disease.

	*N*	RR (95% CI)	*P*_heterogeneity_	*I* ^*2*^ (%)	*P*_differences_
Region					
USA	6	0.97 (0.88–1.08)	0.09	47.3	0.68
Europe	3	1.13 (0.89–1.43)	0.04	68.6	
Sex					
Men	6	1.03 (0.90–1.18)	0.02	64.0	0.73
Women	3	1.04 (0.83–1.30)	0.02	73.4	
Both	5	1.07 (0.90–1.28)	0.02	67.1	
Study duration					
≥15 years	5	1.06 (0.91–1.24)	0.01	70.5	0.30
<15 years	5	0.91 (0.75–1.10)	0.04	60.6	
Age at baseline					
≥60 years	5	0.95 (0.79–1.13)	0.04	60.7	0.51
<60 years	5	1.03 (0.88–1.22)	<0.01	71.5	
No. of cases					
≥200	5	1.05 (0.92–1.20)	0.01	71.3	0.36
<200	5	0.90 (0.72–1.12)	0.05	58.0	
Statistical adjustment					
Age					
Yes	8	0.97 (0.83–1.14)	<0.01	71.7	0.83
No	2	1.01 (0.91–1.14)	0.61	0.0	
Education					
Yes	4	1.11 (0.95–1.30)	0.04	63.9	0.14
No	6	0.92 (0.79–1.06)	0.07	51.5	
Energy intake					
Yes	2	1.03 (0.93–1.15)	0.33	0.0	0.92
No	8	0.98 (0.84–1.14)	<0.01	71.3	
Smoking					
Yes	7	1.00 (0.86–1.16)	<0.01	72.0	0.82
No	3	0.99 (0.84–1.16)	0.21	36.7	
Alcohol consumption					
Yes	4	1.13 (0.99–1.29)	0.08	56.2	0.08
No	6	0.90 (0.78–1.04)	0.08	50.1	
Physical activity					
Yes	5	1.03 (0.85–1.25)	<0.01	75.8	0.50
No	5	0.97 (0.87–1.08)	0.28	21.4	
Coffee consumption					
Yes	6	1.03 (0.88–1.21)	<0.01	70.7	0.47
No	4	0.95 (0.82–1.10)	0.18	39.5	

RR, relative risk; CI, confidence interval.

**Fig 3:**
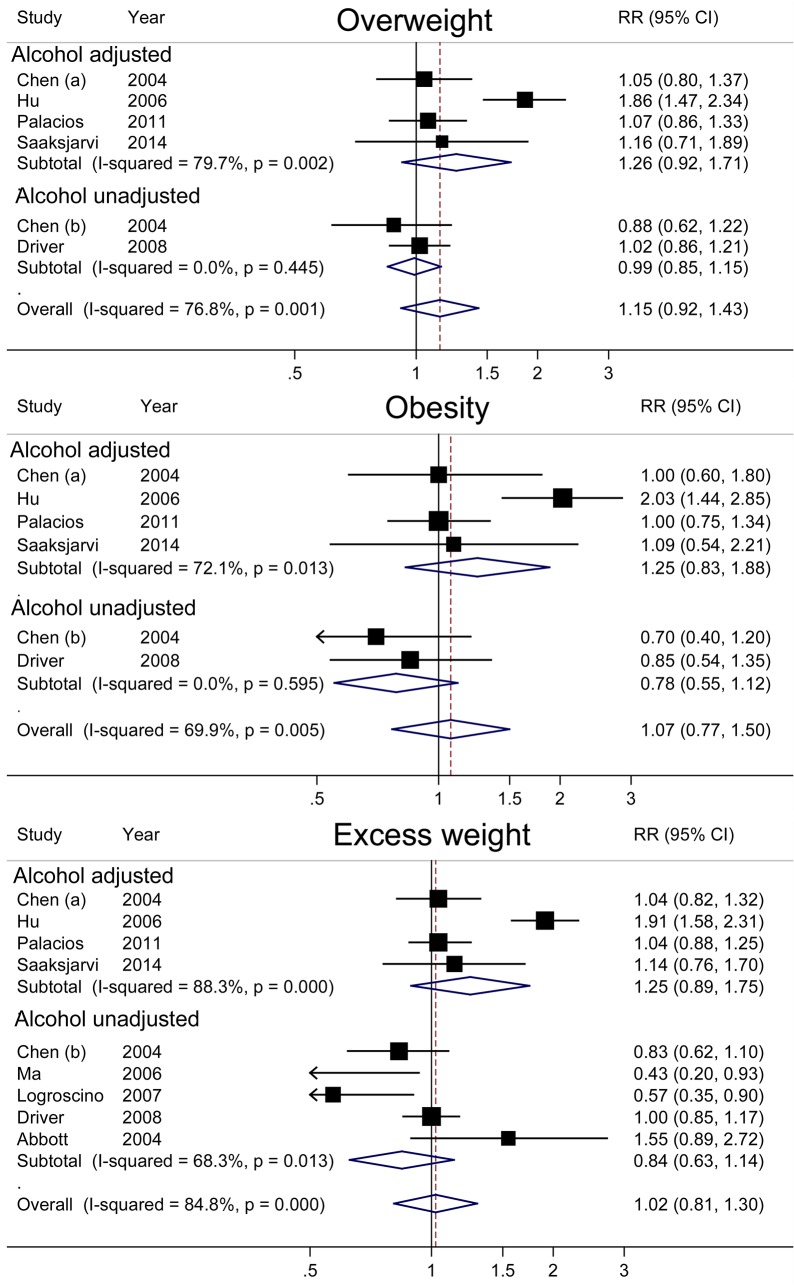
Relative risks and 95% confidence interval of Parkinson’s disease associated with overweight, obesity and excess weight, by adjustment for alcohol consumption.

In the separate analyses that examined the relationship between obesity status and PD risk, the summary RRs were 1.15 (95% CI = 0.92–1.43) for overweight, 1.07 (95% CI = 0.77–1.50) for obesity, and 1.02 (95% CI = 0.81–1.30) for excess weight (Fig 3). In a further analysis by whether or not adjusting for alcohol consumption, the summary RRs for overweight, obesity and excess weight were 1.26 (95% CI = 0.92–1.71), 1.25 (95% CI = 0.83–1.88) and 1.25 (95% CI = 0.89–1.75) in the studies with adjustment for alcohol consumption (Fig 3).

### Publication bias

There was some evidence of publication bias according to Egger’s test (*P*_Egger_ = 0.05), but not Begg’s test (*P*_Begg_ = 0.11).

## Discussion

In this meta-analysis of 10 prospective studies consisting of 2,706 PD cases and 430,854 participants, we showed no association between BMI and risk of PD. Additional analyses investigating obesity status, as defined by BMI levels, in relation to PD risk found similar results.

Significant heterogeneity was observed in this meta-analysis. This may be attributed to the high heterogeneity among included epidemiological studies. These studies were different in population characteristics, study duration, and methodologies (e.g., statistical adjustment for confounders). The differences in adjustment should be of particular consideration, as different populations may have their own pattern of risk factors (e.g., socioeconomic status, diets, and lifestyles) that affect both body composition and PD risk.

We carried out subgroup analysis by variables that were adjusted for in the original studies. Cigarette smoking is a widely reported protective factor for PD[25]. However, similar findings were generated in studies with adjustment for smoking and in those without. We considered that, as a confounding factor, smoking was likely to have exaggerated rather than diluted any positive relationship between BMI and PD due to the fact that smokers (have lower PD risk) generally have a lower BMI than non-smokers. A similar explanation could be applied to physical activity, a factor that is inversely associated with both BMI and PD [10, 26]. We found some evidence of increased PD risk in studies that adjusted for alcohol consumption (RR = 1.13, 95% CI = 0.99–1.29), and decreased risk in studies that did not (RR = 0.90, 95% CI = 0.78–1.04). A recent meta-analysis[9] of observational studies revealed that alcohol reduced PD risk in a dose-response manner. There was evidence that alcohol drinking, especially heavy drinking, lead to a greater weight gain and higher BMI[8]. Thus, a failure to account for alcohol consumption (or some other factors), which may be positively associated with BMI but inversely with PD may mask any positive BMI-PD association, or even lead to an inverse association. Therefore, a week increase in PD risk associated with higher BMI is still possible, and more studies carefully adjusting for potential confounders are needed.

There has been clear evidence that PD patients generally have lower BMI when comparing with healthy controls[3]. Some investigations[4, 5] found that BMI decline started 2–5 years prior to the clinical diagnosis of PD, which indicates that such preclinical weight change may lead to some undiagnosed PD patients and, therefore, mask the true BMI-PD association. Several included cohorts[4, 5, 23, 24] had attempted to exclude the potential influence of preclinical weight change by conducting sensitivity analyses excluding participants who were diagnosed during the first 2–5 years of follow-up, and their findings were not significantly altered. A similar observation was found in the Finish Mobile Clinic Health Examination Survey [10] after excluding the first 10 years of follow-up. However, after the exclusion of 15 years, a positive association between BMI and the occurrence of PD emerged (*P* for trend = 0.02), which indicates that the lag time between weight loss and PD diagnosis may be quit long. We used 15 years as a cut-off point to conduct the subgroup analysis by study duration, but no significant findings were observed.

The null BMI-PD association did not exclude possible associations between other measures of obesity and PD risk. In the Honolulu Heart Program, increased triceps skinfold thickness, an indicator of peripheral obesity, was associated with elevated risk of PD. In two large US cohorts of men and women[4], there was a positive association between waist circumference (WC) and PD risk among never smokers, and in another cohort from the USA[24], higher WC was non-significantly associated with risk of PD (RR = 1.35, *P* for trend = 0.08).

This meta-analysis had several strengths. Including studies with a prospective design eliminated recall and selection biases, both of which are common in retrospective case-control studies. Including a large number of PD case strengthened the statistical power of the current study. Furthermore, the levels of BMI and statistical adjustments across studies were substantially distinct. We conducted the dose-response meta-analysis to make better comparability between studies and carried out several stratified analyses to explore the potential impacts confounders on the BMI-PD association.

However, several limitations should also be acknowledged when interpreting the results of this meta-analysis. Our study is a meta-analysis of observational studies that inherits the weaknesses of the primary studies. Of particular note is the problem of confounders. As was mentioned above, confounding factors may have biased our results toward either direction. In addition, original studies only conducted a single measurement of baseline BMI, and did not take weight changes during the long follow-up duration into account, resulting in some participants being misclassified. Misclassification of exposure in prospective studies would likely to be non-differential, leading to an underestimation of the risk estimates. Furthermore, potential publication bias also merits consideration because small studies with null results are less likely to be published compared with larger studies or studies with positive findings. Despite the presence of such bias, a further inclusion of any studies with null results would be unlikely to change our overall findings.

In sum, findings from this meta-analysis of prospective studies exclude a considerable association between BMI, or obesity status measured by BMI, and the future occurrence of PD. However, a week positive association that may be masked by confounders still cannot be excluded, and future prospective studies with a good control for potential confounding factors are required.

## Supporting Information

S1 PRISMA Checklist PRISMA Checklist. (DOCX)

S1 Table A list of excluded studies with reasons for exclusion. (DOCX)
