# Supplementary material for: Body Mass Index and Risk of Parkinson’s Disease: A Dose-Response Meta-Analysis of Prospective Studies
Source: PLoS One. 2015 Jun 29;10(6):e0131778. doi: 10.1371/journal.pone.0131778 (PMC4488297; doi:10.1371/journal.pone.0131778)
Supplement: S1 Table — (DOCX) [file pone.0131778.s002.docx]

| Study | Year | Reasons for exclusion |
| --- | --- | --- |
| Chen et al.[[1](#_ENREF_1)] | 2005 | No data on body mass index |
| Abbott et al.[[2](#_ENREF_2)] | 2003 | No data on body mass index |
| Barichella et al.[[3](#_ENREF_3)] | 2003 | Case-control study |
| Ragonese et al.[[4](#_ENREF_4)] | 2008 | Case-control study |
| Morales-Briceno et al.[[5](#_ENREF_5)] | 2012 | Case-control study |

**Table S1** A list of excluded full-text articles

**References**

1. Chen H, Zhang SM, Schwarzschild MA, Hernan MA, Ascherio A (2005) Physical activity and the risk of Parkinson disease. Neurology 64: 664-669.

2. Abbott RD, Ross GW, White LR, Sanderson WT, Burchfiel CM, et al. (2003) Environmental, life-style, and physical precursors of clinical Parkinson's disease: recent findings from the Honolulu-Asia Aging Study. J Neurol 250 Suppl 3: III30-39.

3. Barichella M, Marczewska A, Vairo A, Canesi M, Pezzoli G (2003) Is underweightness still a major problem in Parkinson's disease patients? Eur J Clin Nutr 57: 543-547.

4. Ragonese P, D'Amelio M, Callari G, Di Benedetto N, Palmeri B, et al. (2008) Body mass index does not change before Parkinson's disease onset. Eur J Neurol 15: 965-968.

5. Morales-Briceno H, Cervantes-Arriaga A, Rodriguez-Violante M, Calleja-Castillo J, Corona T (2012) Overweight is more prevalent in patients with Parkinson's disease. Arq Neuropsiquiatr 70: 843-846.
